# Residue Asn277 Affects the Stability and Substrate Specificity of the SMG1 Lipase from *Malassezia globosa*

**DOI:** 10.3390/ijms16047273

**Published:** 2015-03-31

**Authors:** Dongming Lan, Qian Wang, Jinxin Xu, Pengfei Zhou, Bo Yang, Yonghua Wang

**Affiliations:** 1 School of Bioscience and Bioengineering, South China University of Technology, Guangzhou 510006, China; E-Mails: dmlan@scut.edu.cn (D.L.); wangqian_0315@126.com (Q.W.); pfzhougz@foxmail.com (P.Z.); 2 State Key Laboratory of Respiratory Disease, Guangzhou Institutes of Biomedicine and Health, Chinese Academy of Sciences, Guangzhou 510530, China; E-Mail: xu_jinxin@gibh.ac.cn; 3 College of Light Industry and Food Sciences, South China University of Technology, Guangzhou 510640, China

**Keywords:** mono- and diacylglycerol lipase, thermostability, substrate selectivity, site-directed mutagenesis

## Abstract

Thermostability and substrate specificity are important characteristics of enzymes for industrial application, which can be improved by protein engineering. SMG1 lipase from *Malassezia globosa* is a mono- and diacylglycerol lipase (MDL) that shows activity toward mono- and diacylglycerols, but no activity toward triacylglycerols. SMG1 lipase is considered a potential biocatalyst applied in oil/fat modification and its crystal structure revealed that an interesting residue-Asn277 may contribute to stabilize loop 273–278 and the 3_10_4 helix which are important to enzyme characterization. In this study, to explore its role in affecting the stability and catalytic activity, mutagenesis of N277 with Asp (D), Val (V), Leu (L) and Phe (F) was conducted. Circular dichroism (CD) spectral analysis and half-life measurement showed that the N277D mutant has better thermostability. The melting temperature and half-life of the N277D mutant were 56.6 °C and 187 min, respectively, while that was 54.6 °C and 121 min for SMG1 wild type (WT). Biochemical characterization of SMG1 mutants were carried out to test whether catalytic properties were affected by mutagenesis. N277D had similar enzymatic properties as SMG1 WT, but N277F showed a different substrate selectivity profile as compared to other SMG1 mutants. Analysis of the SMG1 3D model suggested that N277D formed a salt bridge via its negative charged carboxyl group with a positively charged guanidino group of R227, which might contribute to confer N277D higher temperature stability. These findings not only provide some clues to understand the molecular basis of the lipase structure/function relationship but also lay the framework for engineering suitable MDL lipases for industrial applications.

## 1. Introduction

Lipases (E.C. 3.1.1.3) (Enzyme Committee 3.1.1.3) are unique biocatalysts that catalyze hydrolysis reactions in aqueous conditions and esterification, interesterification, and transesterification reactions in microaqueous media [1]. They have attracted much attention due to their remarkable characterization, such as specific regio- and stereoselectivity, high activity, and stability in non-aqueous environments [2]. Those properties make them of great value for application in industries, such as food, detergent, pharmaceutical, leather, biodiesel, fine chemicals and paper industries [1,2].

Lipases belong to the family of α/β hydrolase fold and most of them contain a lid that controls access of the substrate into the lipase active center. In the presence of hydrophobic interfaces (oil-water mixture) [3], organic solvents [4], hydrophobic surfaces [5,6] or detergents [7], the lid may undergo a conformational change that makes the binding site accessible to the substrate. Binding of substrates to the catalytic cavity requires interactions between the acyl group of acylglycerols and the side chain of residues inside the catalytic cavity. Thus residues forming the catalytic cavity, such as those in the lid region or around active site, were considered as the “hot spots” in protein engineering for altering the enzyme properties, such as catalytic activity, substrate specificity, and thermostability [8,9,10]. Santarossa *et al.* reported that substitutions at residues T137 and T138 in the lid region of *Pseudomonas fragi* lipase resulted in a change of the chain length preference profile and improvement of thermostability [11]. Mutation of residues with a high B factor around the catalytic Ser105 residue of CALB lipase can enhance thermostability dramatically without sacrifice of its activity [12]. The catalytic pocket of lipase from *Pseudomonas aeruginosa* was reconstructed by the combinatorial active-active saturation test (CAST) method to expand the range of substrate acceptance [13]. Besides that, immobilization has been recognized as an alternative approach to modulate the catalytic properties of lipases [14,15]. In our previous study, a mono- and diacylglycerols lipase (MDL) from *Malassezia globosa* (named as SMG1, the first characterized MDL gene from *Malassezia globosa*) was cloned and expressed in *Pichia pastoris* (*P. pastoris*) [16]. SMG1 is able to produce partial acylglycerols (only mono- and diacylglycerols without triacylglycerols) by esterification of glycerol with fatty acid due to its unique substrate selectivity. Diacylglycerols (DAG) with high purity can be obtained by enzymatic digestion of monoacylglycerols following molecular distillation [17]. Therefore, SMG1 lipase was a prospective enzyme for use in production of DAG [16,18]. DAG was considered as a type of healthy cooking oil that can reduce body weight and visceral fat accumulation in rats and humans [19,20]. The crystal structure of SMG1 has been determined at 1.45 and 2.6 Å resolutions, revealing that the catalytic triad (Ser171–Asp228–His281) was shielded by a putative lid loop (Thr101 to Asp119) [21]. To understand the substrate specificity of the SMG1 lipase, a model with open conformation were constructed and docking of substrate toward the catalytic site was conducted, demonstrating that bulk amino acids located in the entrance might hinder the entrance of triacylglycerol (TAG) into the catalytic cave [22]. Through a mutagenesis study, two bulky aromatic residues around the catalytic sites in SMG1, W116 and W229, were found to be involved in substrate recognition and contribute to the thermostability [23]. Furthermore, the deep elucidation of the structure/function relationship of SMG1 lipase can pave the road to engineer a DAG-like lipase more suitable for industrial applications.

In this study, we chose Asn277 of SMG1, a hydrophilic residual on the entrance of catalytic cavity and the opposite side of the lid, as a representative residue to investigate the functions of residues on the rim of catalytic cavity. To determine the role of N277 on effecting the stability and catalytic activity of SMG1, N277 was replaced by amino acids with various properties, such as hydrophobic (Leu and Val), bulky (Phe) and anionic (Asp) amino acids. The mutants were expressed in *P. pastoris* and purified to homogeneity using anion exchange chromatography. The catalytic activity, substrate preference and thermostability of the purified mutants were investigated and the structural molecular basis of SMG1 mutants was discussed.

## 2. Results and Discussion

### 2.1. N277 Stabilizing Loop 273–278 and 3_10_4 Helix

**Figure 1:**
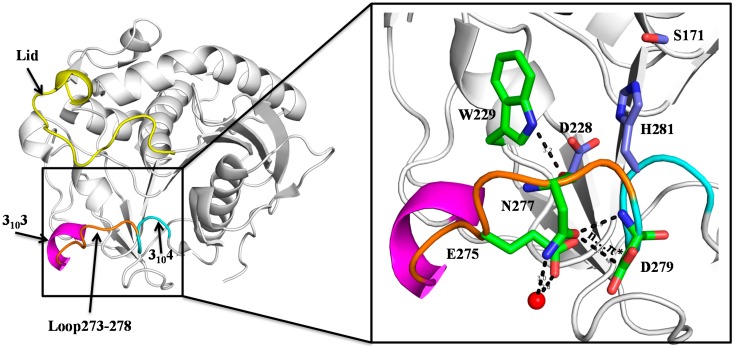
Crystal structure of SMG1 (PDB code: 3UUE, closed form). Lid, 3_10_3 helix, Loop 273–278 and 3_10_4 helix are colored as yellow, magentas, orange and cyan, respectively. The carbon atoms of catalytic triad colored as light blue; the carbon atoms of W229, E275, N277 and D279 colored as green; for all of the displayed residues, oxygen and nitrogen atoms are colored as red and blue, respectively. Water is shown as red sphere.

We reported the crystal structures of SMG1 [21] (PDB ID: 3UUE), which revealed that loop 273–278 connecting two 3_10_ helix, helix-3_10_3 (G268–L272) and 3_10_4 (D279–Q282), is on the opposite site of the putative lid and contributes to catalytic pocket formation. Revisiting the structure of SMG1, we found an interesting residue-N277, which may contribute to stabilize loop 273–278 and 3_10_4 helix. As shown in Figure 1, the side chain of N277 moves away from the catalytic pocket, and interacts with D279 through a hydrogen bond and n–π* interaction. A n–π* interaction formed by the delocalization of the electron pair (n) of a donor group into the antibonding orbital (π*) of a carbonyl group, which is worth about 25% hydrogen bond [24]. Besides interaction with D279, N277 also forms hydrogen bonds with W229 and interacts with E275 through water. With those interaction networks, N277 may contribute to stabilize loop 273–278 and 3_10_4 helix, and influence thermodynamic stability. Since His281 (one of the catalytic triads) is located within the 3_10_4 helix, stabilization of the 3_10_4 helix also supports the correct conformation of His281 during the catalytic process. Therefore we speculate that N277 plays an important role in thermodynamic stability and catalytic activity of SMG1.

### 2.2. Biochemical Properties of SMG1 Mutants

To investigate the role of N277 in the thermodynamic stability and catalytic activity of SMG1, we constructed several mutants: N277D, N277V, N277L and N277F, and studied their biochemical properties.

#### 2.2.1. Effect of Temperature and pH

The effect of temperature on the activities and stability of the SMG1 lipase and its mutants were determined spectrophotometrically using *p*-nitrophenol (pNP) caprate as substrate. The results are shown in Table 1. Comparing with the optimum temperature at 25 °C for wild type SMG1 lipase, the optimal temperature for N277D, N277L, N277F decreased to be 20 °C, and 15 °C for N277V. The specific activity of N277D mutant (16,081.8 U/g) was higher than that of SMG1 WT (15,425.4 U/g), while other mutants showed lower activity as compared to that of SMG1 WT.

**Table 1:** Thermostability and basic properties of SMG1 WT and its mutants.

Mutant	T_opt_ (°C)	pH_opt_	Substrate_opt_	Thermostability (%) ^a^	Specific Activity (U/g) ^b^	*Tm* (°C) ^c^
SMG1 WT	25	6	pNP caprylate	67.7	15,425.4	54.6
N277D	20	6	pNP caprylate	78.7	16,081.8	56.6
N277V	15	6	pNP caprylate	38.9	5292.2	41.1
N277L	20	6	pNP caprylate	33.5	4320.5	42.6
N277F	20	6	pNP caprylate	0	535.2	38.3

^a^ The thermostability was indicated by measured the residual activity of the recombinant lipase after incubating at 40 °C for 1 h. The results showed in this table were averages calculated from at least three independent experiments by spectrophotometric method described in the text; ^b^ Specific activity of purified recombinant lipase to pNP caprylate under optimal temperature and pH of each enzymes; ^c^ The melting temperature of mutants were calculated from the CD data using Global Analysis T-Ramp software (Applied Photophysics, Surrey, UK).

**Figure 2:**
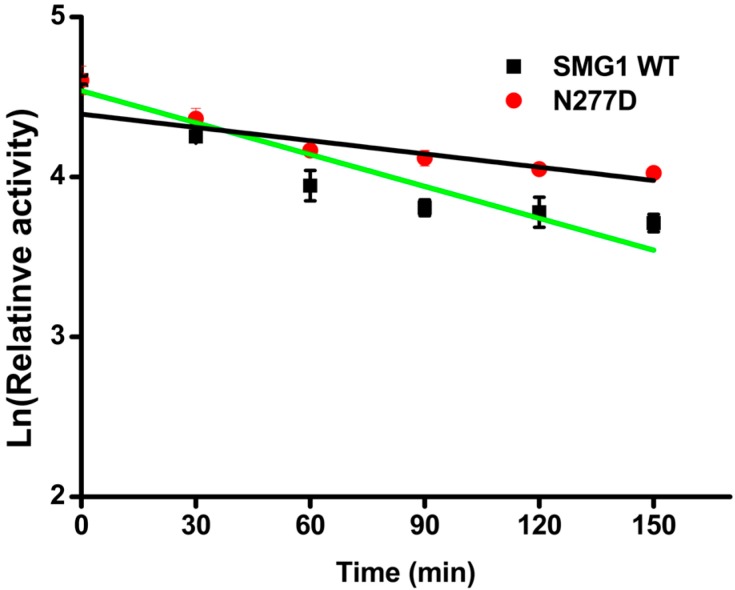
Deactivation of SMG1 WT and N277D. Enzymes were incubated at 45 °C and residual activity was measured every 30 min. The data were fitted to the first-order plots. The half-life of SMG1 (green line) and N227 (black line) were calculated as 121 and 187 min respectively based on the formula of T_1/2_ = ln 2/*k*_d_.

The thermostability of mutants was investigated by incubation at 40 °C for 1 h and the residual activities were then measured. N277D showed a better thermostability than that of SMG1 WT, retaining 78.7% activity of its original activity after incubation. The half-life of N277D was 187 min, while SMG1 was 121 min (Figure 2). N277L and N277V were found to sharply decrease their activity after incubation and only maintained 33.5% and 38.9% of their original activities, respectively. Under the same condition N277F mutant was found to be totally inactivated. CD spectroscopy was employed to investigate the thermodynamic property of SMG1 and its mutants. It is also shown from Table 1 that mutants with hydrophobic amino acids (Val, Leu and Phe) at position 277 increased the instability of protein, while mutants with hydrophilic amino acid (e.g., D) has higher stability by comparing with the SMG1 WT. The melting temperature (*Tm*) for N277D was 56.6 °C which is 2 °C higher than that of SMG1 WT (54.6 °C). But substitution of 277Asn with Phe caused a significant decrease in the *Tm* (38.3 °C), which was consistent with results of thermostability described above. But the replacement of N277 with Asp, Val, Leu and Phe did not led to significant change of secondary structure contents in comparison with that of SMG1 WT (Table S1 in Supplementary Materials).

The effect of pH on the activity and the stability of SMG1 and mutants were studied. All enzymes showed highest activity at pH 6. The enzyme stability was tested after incubation at different pH value buffers for 12 h. The SMG1 WT was found to be stable over a range of pH from 4 to 9, while mutants were stable in buffer solution of pH 7–9 (Figure S1 in Supplementary Materials).

####  2.2.2. Substrate Specificity of Artificial pNP Esters

**Figure 3:**
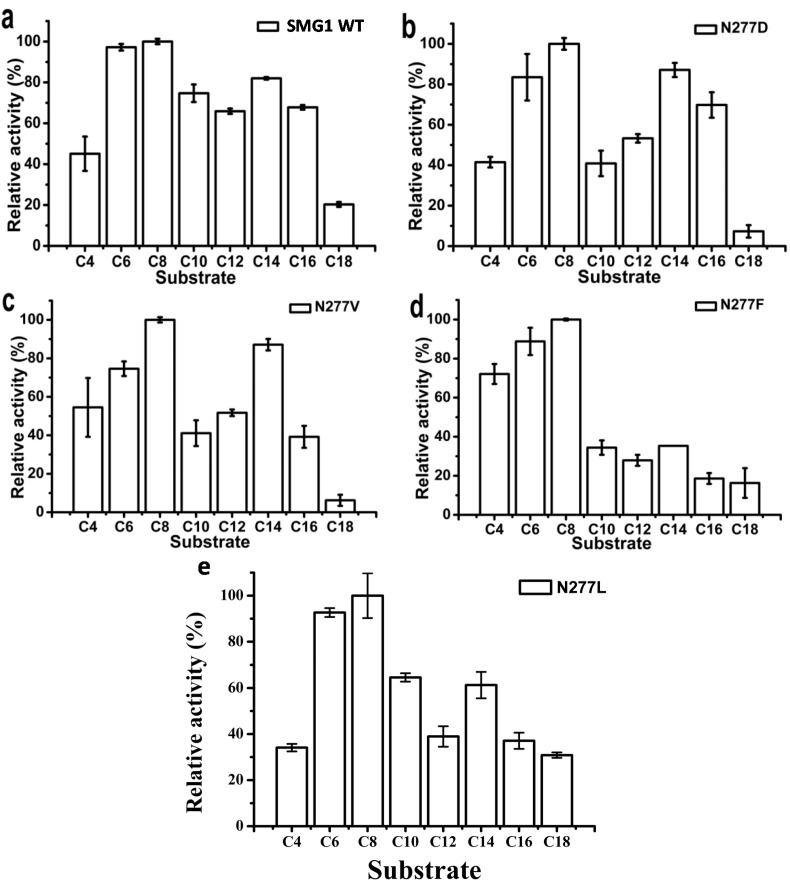
Substrate-specificities of SMG1 and its mutants toward various pNP esters. The relative activity is expressed by using the highest activity of each sample as 100%, and all other values have been standardized to this reference value. (**a**) SMG1 WT; (**b**) N277D; (**c**) N277V; (**d**) N277F; (**e**) N277L.

pNP esters with various carbon chain lengths were used to investigate the substrate preference of SMG1 WT and its mutants. As shown in Figure 3, the optimal substrate for all the enzymes tested was pNP caprylate. SMG1 WTshowed a small difference in reactivity varying with the chain length of pNP esters (C6–C16), while N277F mutant showed a clear preference for short- and medium-chain length pNP esters (C4–C8) (Figure 3d). Substitution with Phe will increase the bulkiness of the amino acid at position 277 since it contains a unique benzene ring structure. The steric hindrance of Phe will affect the access of long-chain substrates to the catalytic pocket. N277D, N277L and N277V displayed similar specificity spectrum.

The specific activities of the purified enzymes were determined using pNP caprylate as substrate. Specific activities of the SMG1 mutants with hydrophobic amino acids (Val, Leu and Phe) substitution decreased considerably compared with that of SMG1, while there was an increase in specific activity of N277D (Table 1).

#### 2.2.3. Substrate Specificity of Acylglycerols

**Figure 4:**
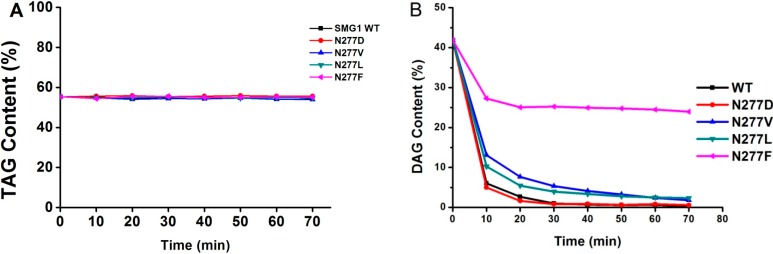
Time course of hydrolysis of acylglycerols (mixture of TAG and DAG) by SMG1 and its mutant. Reaction conditions are as follows: Enzyme load, 6.7 U/g of acylglycerols substrate; Temperature, 25 °C; Reaction time, 70 min; Water, 25% with respect to total reactants; Agitation speed, 200 rpm; (**A**) TAG contents; (**B**) DAG contents.

For acylglycerol substrate, DAG-rich oil harboring 54.52% of TAG and 41.42% of DAG was used as substrate for determination. All SMG1 mutants were inactive towards TAG (Figure 4A) but were able to hydrolyze DAG (Figure 4B), indicating that the locus 277 may not be a key residue involving in substrate recognition between TAG and DAG. As shown in Figure 4B, DAG was completely hydrolyzed by SMG1 mutants after a 7 h reaction, while only 17.88% of DAG was hydrolyzed by N277F. Phenylalanine is a bulky amino acid containing a benzyl side chain. N277F is located at the rim of the catalytic pocket, and the entrance of substrate with longer acyl chains into the catalytic site might be affected due to the steric effect of N277F. Thus it might lead to the decrease of the catalytic efficiency of N277F.

The ratio of decreased amount of 1,3-DAG to that of 1,2-DAG at the end of the reaction was calculated and listed in Table 2. Comparing with the Δ1,3-DAG/Δ1,2-DAG for SMG1 WT, the tested enzymes, such as N277D, N277V and N277L, showed a similar ratio ranging from 1.91 to 2.08 after 70 min reaction, while the ratio Δ1,3-DAG/Δ1,2-DAG for N277F is 1.3. The results indicate that replacement of Asn 277 with Phe led to the change of SMG1 WT regiospecificity towards 1,3-DAG and 1,2-DAG.

**Table 2:** The ratio of decreased amount of 1,2-DAG and 1,3-DAG in the hydrolysis reaction.

Mutants	Δ1,3-DAG (%)	Δ1,2-DAG (%)	Δ1,3-DAG/Δ1,2-DAG
SMG1 WT	27.25	14.37	1.90
N277D	27.09	14.18	1.91
N277V	26.68	13.35	2.00
N277L	26.73	12.83	2.08
N277F	10.1	7.78	1.30

#### 2.2.4. Fatty Acids Selectivity of Mutants

The fatty acids specificities profile of SMG1 WT and its variants were studied by analyzing the competition factors of fatty acids with various carbon chain lengths in an esterification reaction. The specificity constants (1/α) of each fatty acids are displayed in Table 3. The 1/α values of SMG1 WT towards C10 to C18 fatty acid were higher than 0.8, demonstrating than SMG1 WT showed preference towards medium and long chain length of fatty acids (C10:0–C18:0). Similar preference patterns for different fatty acids were found for N277D and N277V. N277F showed a different substrate preference profile as compared with SMG1 WT. The 1/α value of C10:0 is 2-fold over that of C18:0, indicating that N277F preferred medium chain length of fatty acids and discriminated those with the long chain length. N277L displayed a high preference for C6:0 and C16:0 fatty acids whose 1/α value were higher than 0.9.

**Table 3:** The specificity constants (1/α) of each fatty acid.

Fatty Acids	SMG1 WT	N277D	N277L	N277V	N277F
C6:0	0.28	0.28	0.91	0.22	0.40
C8:0	0.64	0.64	0.59	0.29	0.94
C10:0	0.91	0.90	0.50	0.71	1.00
C12:0	0.82	0.83	0.46	1.00	0.85
C14:0	0.95	0.96	0.55	0.90	0.64
C16:0	1.00	1.00	1.00	0.98	0.72
C18:0	0.97	0.97	0.81	0.97	0.52

### 2.3. Molecular Basis of SMG1 Mutants

It is well established that hydrophobicity, van der Waals packing, hydrogen bonds, salt bridges and π interactions make most contributions to protein stability [25,26,27]. In SMG1 WT structure, N277 forms hydrogen bonds with an indolyl group of W229 and amide group of D279 and interacts with E275 through water (Figure 1). A n–π* interaction also has been found between two carbonyl groups of N277 and D279. Therefore N277 has been considered as an important residue stabilizing 3_10_4 helix and the catalytic His281 during the catalytic process. Mutants at N277 also have been found to affect the thermodynamic stability and catalytic activity of SMG1.

**Figure 5:**
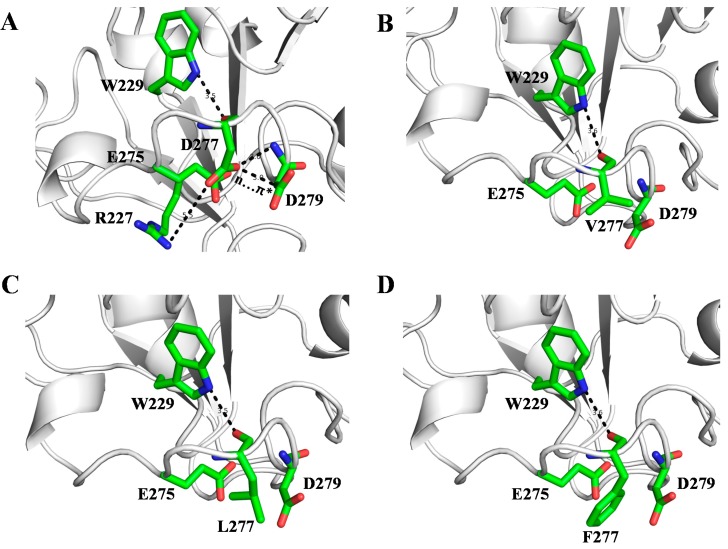
Structure models of N277D (**A**); N277V (**B**); N277L (**C**) and N277F (**D**) mutants. Carbon, oxygen and nitrogen atoms are colored as green, red and blue, respectively.

To better understand how those mutants affect thermodynamic stability and catalytic activity of SMG1, we modeled the 3-D structure of the mutants using the Discover studio V3.5. For the N277D mutant, as Asp and Asn show similar polarity and side chain length, in the structure of N277D mutant D277 also interacts with W229 and D279 (Figure 5A). In comparison with SMG1 WT, N277D mutant contained an additional weak salt bridge which formed by the negative charged carboxyl group of D277 and positive charged guanidino group of R227 (Figure 5A). Salt bridges are an important force for native protein stability [27]. The salt bridge interaction between D277 and R227 may contribute to stabilize SMG1. This finding coincides well with our experimental data that the thermodynamic stability and catalytic activity of SMG1 and the N277D mutant were improved.

In the structures of the N277V, N277L and N277F mutants, hydrophilic interactions of N/D277–E275 and N/D277–E279 were interrupted (Figure 5B–D). In wild type SMG1, N277 is on the surface of the protein and well exposed to the surrounding solvent. In those three mutants, hydrophobic residues V277 and L277 and F277 were also exposed to solvent, and failed to form effective hydrophobic interactions with surrounding residues. Large hydrophobic residues exposed to water will increase side chain conformational entropy, which is a major force for destabilizing proteins [28]. Therefore, replacement of N277 with Val, Leu or Phe, will increase instability of SMG1 and even decrease catalytic activity of SMG1 significantly. This finding also coincides well with our experimental data that the thermodynamic stability and catalytic activity of those three mutants (N277V mutant, N277L mutant and N277F mutant) were significantly reduced. As Phe contains the largest hydrophobic side chain, the N277F mutant showed the lowest thermodynamic stability and catalytic activity compared with the N277V and N277L mutants.

## 3. Experimental Section

### 3.1. Bacterial Strains, Chemicals, Plasmids and Medium

*Escherichia coli* DH5α and plasmid pGAPZαA (Invitrogen, Carlsbad, CA, USA) were used for cloning experiments and *P. pastoris* X-33 strain (Invitrogen) was used as the expression host. The *p*-nitrophenol and pNP esters were purchased from Sigma-Aldrich. Seven fatty acids (FAs): caproic (C6:0, >98%), caprylic (C8:0, >98%), capric (C10:0, >98%), lauric (C12:0, >98%), myristic (C14:0, >98%), palmitic (C16:0, >97%) and stearic (C18:0, >98%) acids were purchased from Aladdin Chemistry Co., Ltd. (Shanghai, China). *n*-Hexane and 2-propanol of HPLC grade were from Kermel Chemical Reagent (Tianjin, China). The DAG-rich oils (54.52% of TAG, 41.42% of DAG and 2.63% of Fatty acids) were prepared by hydrolysis of rape oil using Palatase 20000L (Novo Nordisk A/S, Bagsvaerd, Denmark) and purification by molecular distillation in our lab. All other chemicals were of analytical grade.

Site-directed mutagenesis of SMG1 gene was carried out by the overlapping extension method and primers used for this study were listed in Table S2 in the Supplementary Materials. The PCR products of mutated SMG1 gene were digested with *Kpn* I and *Sal* I and cloned into pGAPZαA. All the constructions were confirmed by DNA sequencing and linearized by restriction enzyme *Bln* I and transformed into *P. pastoris* X-33 strain by electroporation. The transformants were grown on the YPD plates containing Zeocin™ (100 mg/mL) at 30 °C until colonies form. The Zeocin-resistant clones with high expression level were selected for expression.

### 3.2. Expression and Purification of SMG1 and Its Mutants

The recombinant strains were grown in YPD medium (1% (*w*/*v*) yeast extract, 2% (*w*/*v*) peptone and 2% (*w*/*v*) glucose) in a 500 mL glass flask (30 °C, 200 rpm, 72 h). The fermentation broth was centrifuged at 10,000× *g* for 20 min at 4 °C. The pellet was discarded and the supernatant was filtered through a 0.45 µm filter membrane by suction filtration, then concentrated and buffer-exchanged to buffer A (20 mM Tris-HCl, pH 8.0, at 4 °C) using Vivaflow 200 (Vivascience, Hannover, Germany) ultrafiltration membranes with a 10-kDa molecular mass cutoff. The buffer A containing crude enzyme was loaded into a Q Sepharose column and washed with a linear ion gradient (0–200 mM NaCl in buffer A). The fraction containing active protein was concentrated by ultrafiltration and exchanged with 0.1 M phosphate buffer (pH 6.0). Parts of the purified enzymes were lyophilized and the lyophilized powder was stored at 4 °C for esterification assays.

Concentration of the purified enzymes was measured using BCA Protein Assay Kit from Sangon Biotech (Shanghai, China) according to the manual from the manufacturer. The purified enzymes were analyzed by 12% sodium dodecyl sulfate (SDS)–polyacrylamide gel electrophoresis (PAGE).

### 3.3. Biochemical Properties of Recombinant Lipases

Lipase activity assay was measured by colorimetric method using pNP esters as the substrate as described by Gao *et al.* [23]. One unit of enzyme activity is defined as the amount of enzyme releasing 1 μmol of pNP per minute. All of the measuring results were the mean of at least triplicate measurements.

To determine the optimal temperature, lipases activity was measured at temperature ranging from 5 to 40 °C at 5 °C intervals using pNP caprylate as substrate. Thermostability of those lipases was determined by pre-incubating them in 40 °C for 1 h and the residual activity were measured at optimal temperature. The deactivation constant *k*_d_ was calculated from the slope of the plot In (residual activity) *versus* time. Formula of T_1/2_ = ln 2/*k*_d_ was used for calculating the half-life of SMG1 and N227.

Optimum pH for the purified lipase was tested by assaying these enzymes in buffers of different pH ranging from 3 to 8. Buffer using in this study were listed below: pH 3: 0.2 M Na_2_HPO_4_–0.1 M citric acid, pH 4–5: 0.1 M sodium citrate–0.1 M citric acid, pH 6–7: 0.1 M phosphate buffer, pH 8: 0.05 M Tris-HCl. The pH stability of those lipase were assayed by pre-incubating those enzymes in buffers of various pH (pH 3–9) for 12 h at 4 °C, and then the residual activities were tested under optimal temperature and pH.

Substrate specificity for SMG1 WT and its mutants was investigated under the standard assay conditions using pNP esters with different chain length (C4–C18 acyl groups) at 1 mM. pNP esters using in this study included pNP butyrate (C4), pNP caproate (C6), pNP caprylate (C8), pNP caprate (C10), pNP laurate (C12), pNP myristate (C14), pNP palmitate (C16) and pNP stearate (C18).

### 3.4. Circular Dichroism Spectral Analysis

Thermal unfolding of SMG1 WT and mutants in sodium phosphate buffer (20 mM phosphate buffer, pH 6.0) were monitored by a chrascan spectropolarimeter (Applied Photophysics, Surrey, UK). The concentration of the tested enzymes was about 0.2 mg/mL. The warm-up period was from 20 to 70 °C with a heating rate of 1 °C·min^−1^. Wavelength of 190 to 260 nm was scanned. Each spectrum was the average of three successive scans. For all measurements, a reference sample containing the corresponding buffer was subtracted from the CD signal. The *Tm* values were calculated from the spectra using Global 3™ Analysis software (Applied Photophysics, Surrey, UK).

### 3.5. Hydrolysis of Acylglycerols

DAG-rich oil is composed of 54.52% of TAG and 41.42% of DAG, and it is used as a reaction substance. The reaction was performed in a 15 mL conical flask with stirring at 200 rpm. The reaction mixture includes 25% of water content with respect to total reactants, enzyme load (8 U/g, with respect to oil), temperature (25 °C) were used to investigate the ability of SMG1 and its mutants on hydrolysis of DAG-rich oil.

Aliquots (150 μL) of the reaction mixture were periodically withdrawn from the reactions and then were centrifuged at 10,000× *g* for 3 min to remove the water from the upper layer. Supernatants (20 μL) were diluted in 1 mL of *n*-hexane/2-propanol/methanoic acid (15:1:0.003 by volume) for HPLC analysis as described by Wang *et al.* [29]. The experiments were performed in triplicate and the results were presented as the average.

### 3.6. Fatty Acid Selectivity Determination

#### 3.6.1. Esterification

The fatty acid selectivity of SMG1 and its mutants were evaluated in a multi-competitive substrate reaction system containing equimolar quantities of various FAs (C6:0–C18:0). A mixture including caproic acid (C6:0), caprylic acid (C8:0), capric acid (C10:0), lauric acid (C12:0), myristic acid (C14:0), palmitic acid (C16:0), stearic acid (C18:0) at equimolar quantities was prepared according to the methods described by Qin *et al.* [30]. Reactions containing 43.19 mmol glycerol, 10.80 mmol equimolar quantities of the FAs, 5% of water (with respect to total reactants, 0.1 M phosphate buffer, pH 6) and appropriate amount of SMG1 or each mutant were carried out in a conical flask (10 mL) with an agitation speed of 200 rpm at 30 °C. The samples were collected and centrifuged at a speed of 16,000 rpm for 10 min, and the acylglycerols and unesterified FAs in the upper phase was obtained and stored for the next procedure. The degree of conversion (C_n_) for each fatty acid (%) was calculated as follows:

(1) $${\text{C}}_{\text{n}}=\frac{\text{A}{\text{V}}_{0}-\text{A}{\text{V}}_{t}}{\text{A}{\text{V}}_{0}}\times F_{n}$$

where AV_0_ and AV*_t_* were acid values of the samples at time zero and time *t*, respectively. *F_n_* was the fatty acid composition of acylglycerols determined by GC.

#### 3.6.2. Gas Chromatography Analysis

Samples containing acylglycerols (MAG and DAG) and unesterified FFA from the esterification assay were separated by thin layer chromatography. The bands were sprayed with 0.2% 2,7-dichlorofluorescein in methanol and visualized under ultraviolet light. The bands of DAG and MAG components were scraped off and treated with KOH solution to convert the acylglycerols to FAMEs according to a method described by Zeng *et al.* [31] with some modification*s.* In short, samples containing acylglycerols was loaded into a 50 mL flask with the addition of 5 mL of 0.5 mol/L methanolic sodium hydroxide solution and 3 mL of methanolic trifluoride solution, and sample was boiled and swirled at 70 °C for 10 min. The upper phase was collected after centrifugation (16,000 rpm for 15 min) for GC analysis.

Agilent 7890A GC (Agilent, Santa Clara, CA, USA) equipped with a capillary column CP-Sil 88 (60 m × 0.25 mm × 0.2 m; Dikma Technologies, Beijing, China) and a flame ionisation detector were used to analyze the FAMEs prepared above. The flow rate of carrier gas (nitrogen) was 1.1 mL·min^−1^. The initial column temperature was 140 °C and was kept for 5 min, raised to 220 °C at 4 °C/min, and maintained at this temperature for 15 min. The temperatures of the injector and detector were set as 250 and 280 °C, respectively. The correction factor values of FAMEs were used to obtain the relative content of FAMEs expressed as molar%.

#### 3.6.3. Determination of the Competitive Factor

The competitive factor (α) for various fatty acids in esterification assay was determined using Equation (2) described by Rangheard *et al.* [32]. Where *A*_0_ and *B*_0_ are the concentrations of the substrate *A* and substrate *B* at time zero, respectively; *A_t_* and *B_t_* are those at time *t*, respectively.

(2) $$\text{α}=\frac{\text{ log (}A_{0}/A_{t})}{\text{ log (}B_{0}/B_{t})}$$

In this study, we used Equation (3) which is derived from Equation (2) for calculating the competitive factor (α). *C*_ref_ and *C*_n_ are the degree of conversion (Equation (1)) of the reference fatty acid and the fatty acid of interest, respectively, and the optimal substrate was taken as the reference fatty acid (α = 1). The specificity constant for a FA was expressed as 1/α.

(3) $$\text{α }=\frac{\text{ log (1 - }C_{\text{ref}})}{\text{log (1- }C_{\text{n}})}$$

### 3.7. 3-D Structure Models of SMG1 Mutants

In order to provide structural insight into catalytic property change of SMG1 mutants, 3-D structure models of mutants were predicted with Discover studio V3.5 (Accelrys, San Diego, CA, USA) based on crystal structure of wild type SMG1 (PDB ID: 3UUE). The modeled 3-D structures of SMG1 mutants were visualized and analyzed using the PyMOL software [33].

## 4. Conclusions

In this study, the biochemical properties of SMG1 can be altered by mutagenesis of N277. We found that thermostability of SMG1 lipase was improved without sacrificing its activity by mutating Asn277 to Asp, while replacement with hydrophobic residues led to increase the instability of SMG1 WT. Mutagenesis of Asn 277 with Phe led to the change of SMG1 regiospecificity towards 1,3-DAG and 1,2-DAG and substrate specific profiles. N277F showed preference towards pNP esters with shorter carbon length, and fatty acids with medium chain length (C8:0, C10:0 and C12:0) in the esterification assay. Although the thermostability is a bottleneck to limit the further application of SMG1 mutants, the obstacle can be overcome via immobilization of enzyme on suitable resins [34]. In future study, mutagenesis of N277 by amino acids with various sides chains (electrically charged or polar uncharged) will be performed to fully understand its function on SMG1 lipase, which might shed light on the structural and functional relationship of MDLs.

## Supplementary Materials

Supplementary materials can be found at http://www.mdpi.com/1422-0067/16/04/7273/s1.
