# Supplementary material for: Residue Asn277 Affects the Stability and Substrate Specificity of the SMG1 Lipase from Malassezia globosa
Source: Int J Mol Sci. 2015 Mar 31;16(4):7273–88. doi: 10.3390/ijms16047273 (PMC4425016; doi:10.3390/ijms16047273)
Supplement: Supplementary file 1 [file ijms-16-07273-s001.pdf]

Supplementary Information

Table S1. The contents of secondary structure of SMG1 WT and its mutants.

Secondary Structure	SMG1 WT	N277D	N277L	N277V	N277F
Helix	6.60%	6.90%	7.30%	7.00%	7.30%
Antiparallel	33.80%	31.70%	31.90%	30.40%	35.80%
Parallel	3.20%	3.20%	3.20%	3.10%	3.40%
Beta-Turn	21.20%	22.00%	22.70%	23.00%	21.10%
Rndm. Coil	34.90%	35.70%	35.20%	36.10%	33.60%

Table S2. Primers used for construction of SMG1 lipase mutants.

Name	Primer Sequences [a]
SMG1 For	5'-GGGGTACCAGCAGTATTTACGCCCGTGGCCG-3'
3'AOX	5'-GGCAAATGGCATTCTGACAT-3'
N277F For	5'-GCTCGCGAGTTCTTCTTTGACG-3'
N277F Rev	5'-CGTCAAAGAAGAACTCGCGAGC-3'
N277D For	5'-GCTCGCGAGTTCGACTTTGACG-3'
N277D Rev	5'-CGTCAAAGTCGAACTCGCGAGC-3'
N277V For	5'-GCTCGCGAGTTCGTCTTTGACG-3'
N277V Rev	5'-CGTCAAAGACGAACTCGCGAGC-3'
N277L For	5'-GCTCGCGAGTTCCTTTTTGACG-3'
N277L Rev	5'-CGTCAAAAAGGAACTCGCGAGC-3'

[a] Mutations introduced are underlined.

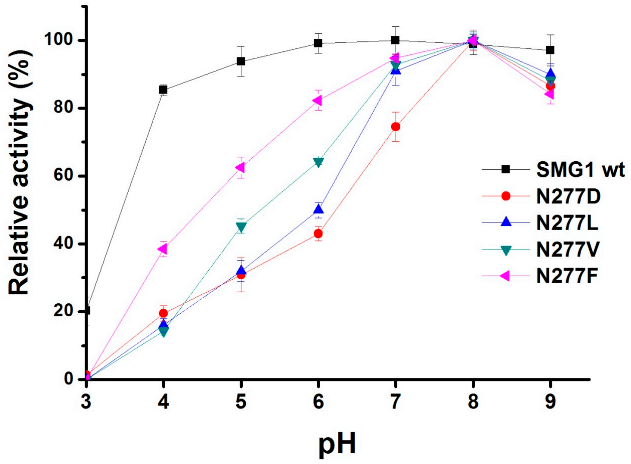

Figure S1. Effects of pH on the stability of SMG1 WT and its mutants. Enzymes tested were incubated in the buffers with various pH ranging from 3 to 9, and the residual activities were measured at 25 °C and pH 6. The relative activities were calculated by using the highest activity of each sample as 100%.
